# Review of EEG-based pattern classification frameworks for dyslexia

**DOI:** 10.1186/s40708-018-0079-9

**Published:** 2018-06-15

**Authors:** Harshani Perera, Mohd Fairuz Shiratuddin, Kok Wai Wong

**Affiliations:** School of Engineering and Information Technology, Murdoch University, Murdoch, Australia

**Keywords:** Dyslexia, Electroencephalogram, Feature extraction, Artefact removal, Artefact subspace reconstruction, Support vector machine, Classification

## Abstract

Dyslexia is a disability that causes difficulties in reading and writing despite average intelligence. This hidden disability often goes undetected since dyslexics are normal and healthy in every other way. Electroencephalography (EEG) is one of the upcoming methods being researched for identifying unique brain activation patterns in dyslexics. The aims of this paper are to examine pros and cons of existing EEG-based pattern classification frameworks for dyslexia and recommend optimisations through the findings to assist future research. A critical analysis of the literature is conducted focusing on each framework’s (1) data collection, (2) pre-processing, (3) analysis and (4) classification methods. A wide range of inputs as well as classification approaches has been experimented for the improvement in EEG-based pattern classification frameworks. It was uncovered that incorporating reading- and writing-related tasks to experiments used in data collection may help improve these frameworks instead of using only simple tasks, and those unwanted artefacts caused by body movements in the EEG signals during reading and writing activities could be minimised using artefact subspace reconstruction. Further, support vector machine is identified as a promising classifier to be used in EEG-based pattern classification frameworks for dyslexia.

## Introduction

Dyslexia is a disability that involves deficiencies in reading and writing capabilities, but does not affect intellect. Although this condition was commonly known as ‘word blindness’ in the 1800s, it has now been identified as a condition with a neurological origin and not as a condition to do with lack of vision [1, 2].

There are many techniques proposed by past research to detect indicators of dyslexia. We can broadly categorise these techniques into three. The first category is detection using ‘behavioural’ symptoms and aspects. This is the conventional and most popular method that is currently used by psychologist to diagnose dyslexia. This method assesses whether a person has dyslexia using highly recognised standardised tests [3]. The second category is the use of brain imaging techniques to portray distinctive brain behaviours [4]. Functional magnetic resonance imaging (fMRI), magneto-encephalography (MEG), electroencephalography (EEG) and positron emission tomography (PET) are few of the methods that could be used to depict these behaviours. Studies show [4–6] that individuals with dyslexia have unique brain structures and behaviours. The third category includes eye-movement patterns [7–9]. Category two and three are still in experimental stages. Those techniques only help to identify symptoms of dyslexia and are not currently used to diagnose dyslexia.

EEG is one of the popular techniques used to assess brain behaviours. In this study, we look at how EEG has been used to identify signs of dyslexia. The EEG results may help psychologist to complement the current dyslexia assessing techniques as it could add a neurological point of view.

This paper focuses on EEG-based dyslexia studies that have attempted to identify unique brain activations using pattern recognition. Although dyslexia can further be divided into sub-types, this study does not cover studies that use pattern recognition to identify the sub-types. The intension of this study is to review EEG-based pattern classification frameworks specific to dyslexia and do not intend to speculate the neuroscience behind the findings. Each framework is assessed using a pre-defined format to arrange the data in a meaningful manner and to recognise its strengths and weaknesses. These discoveries are then used to propose an improved EEG-based pattern classification framework for dyslexia (higher validation accuracies for the classifier).

### What is dyslexia?

Dyslexia is a disability with a neurological origin that causes difficulties in reading, writing or spelling despite average or above average intelligence and sensory abilities. Common symptoms of dyslexia include poor reading skills, unreadable handwriting, slow writing or copying, bad spellings, letter migration or reversals. [2, 10–13].

This condition is heritable, which means that a child might inherit it from a parent. It has been reported that 23–65% of children who have a parent with dyslexia are at risk of having dyslexia [14]. Dyslexia in some cases can have partly or wholly distinct genetic causes. Studies [15] suggest looking into the genetic aspect to effectively detect dyslexia instead of merely considering individual disabilities. Studies have shown that overall reading capabilities including dyslexia have noteworthy genetic components with heritability estimated at 54–84% [16]. Left-handedness is sometimes considered to be prevalent among people with dyslexia. However, there seems to be a controversy, certain findings have discovered a connection between dyslexia and left-handedness, whereas some studies claim it to be a myth [17–19].

### Why is dyslexia detection important?

A noteworthy amount of the world population is affected by dyslexia. Statistics show that approximately 20% of the child population in the USA [20], approximately 4% of the students in Australia [21] and overall approximately 15–20% of the world population [22] experience dyslexia.

An individual with dyslexia can become a depressed, unmotivated or a low self-esteemed if the condition goes undetected. Difficulty in learning to interpret letters, words or sometimes even symbols certainly causes the child to have a hard time keeping up with peers [4, 23].

Diagnosing dyslexia at an early stage is important to prevent the child having to go through a stressful, rough childhood and face frustrating experiences at school. Early detection helps to direct a child with dyslexia to the necessary treatments required. Targeted assistance is essential for people with dyslexia to cope up with their struggles and difficulties. Recent studies [10] state that ‘dyslexia is not a disease or defect that can be cured’, rather a ‘condition that can be helped’ with proper targeted support. Promising results have shown of children who go through such intervention programs in the early stages [24] proving improvement in reading performance as well as reduction in anxiety [25]. Though these techniques help, dyslexia still does persist into adulthood [26].

Although persons with dyslexia face difficulties in reading and writing, they have normal or sometimes even higher intelligent levels. Albert Einstein, Leonardo da Vinci, Alexander Graham Bell, Hans Christian Andersen, Walt Disney, Henry Ford, Steve Jobs and Richard Branson are few of the famous and talented dyslexic great minds [27]. According to Davis [27] in the book ‘The gift of dyslexia: why some of the brightest people can’t read and how they can learn’, people with dyslexia are believed to be highly intuitive and insightful with the ability to alter and create perceptions. They are known to be highly aware of the environment, with more curiosity than average, thinking mainly in pictures instead of words and experiencing thought as reality with a lot of vivid imaginations [27].

### Conventional dyslexia detection techniques

The conventional dyslexia detection practices are often based on ‘behavioural’ symptoms and aspects [28]. Standardised test such as Wechsler Individual Achievement Test (WIAT), Comprehensive Test of Phonological Processing (CTOPP), Oral and Written Language Scales (OWLS) and Woodcock Johnson (WJ) are used to assess reading, writing, intelligence quotient and phonological processing abilities. The results of the standardised test along with factors such as biographical information and family history help determine whether a person has dyslexia [21]. The severity of dyslexia may vary from mild to severe, and the symptoms of dyslexia vary from person to person [29].

## Electroencephalogram (EEG)

### What is an EEG?

**Fig. 1:**
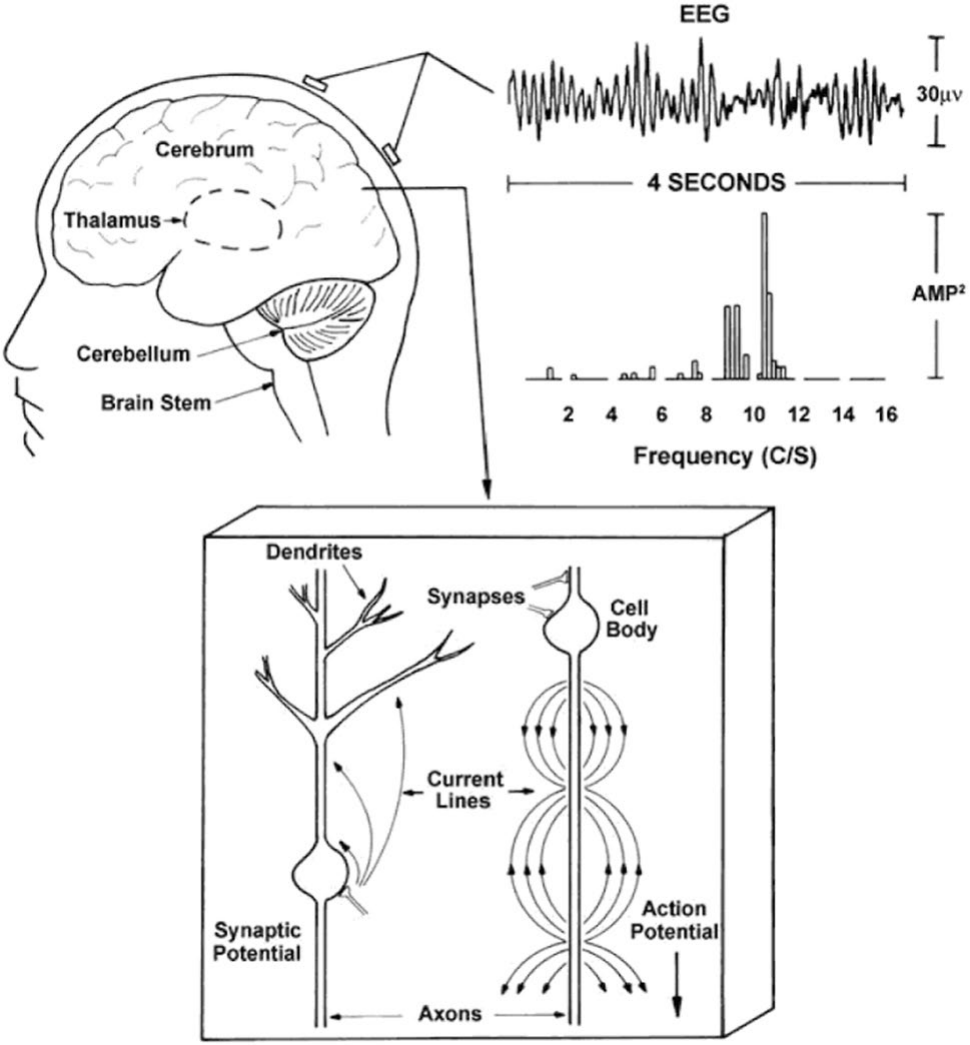
Capturing EEG [30, p. 5]

Electroencephalogram, commonly known as EEG, is a ‘record of the oscillations of brain electric potential recorded from electrodes on the human scalp’ [30, p. 3]. EEG is a technique that can be used to monitor and detect brain functions. The electrical activity of the brain for various stimuli can be identified via the electrodes placed on the scalp (Fig. 1).

### EEG-based pattern classification for dyslexia

EEG is often used for detecting conditions in the brain such as epilepsies, seizures, brain tumours and sleeping disorders [31–36].

Recent studies show that researchers are now looking into ‘neurological’ aspects to identify patterns that are unique to dyslexia. It has been uncovered that there are structural differences as well as different forms of processing of the brain between normal and dyslexic individuals. Dyslexics’ brain is normal and healthy; it takes a longer time to make connections compared to normal people [4, 37].

Similar to other conditions, EEG can also be used to identify unique brain activation patterns of dyslexia since it has a neurological origin. The next section will discuss the efforts made by research in using EEG for pattern classification between dyslexics and non-dyslexics.

## What are the existing frameworks and their shortcomings?

This section covers research carried out to improve pattern classification frameworks for dyslexia using EEG. The existing frameworks will be identified, and each framework will be explored in depth to identify its strengths and weaknesses.

**Fig. 2:**
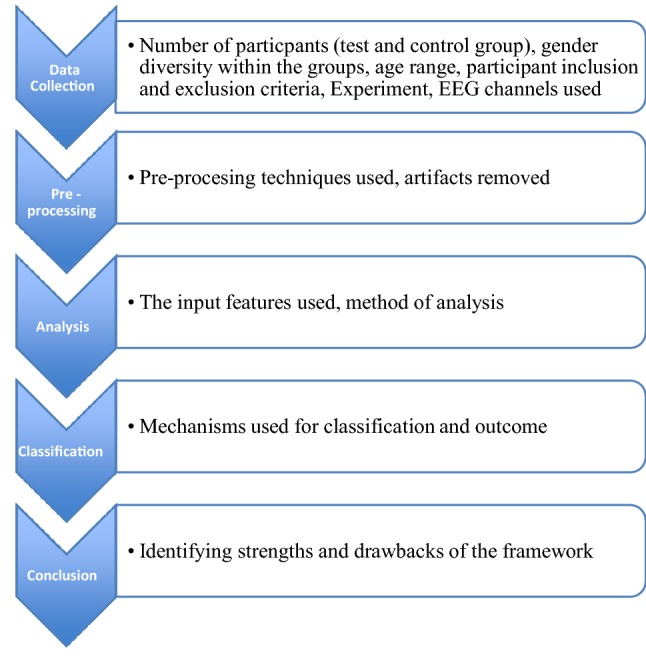
Overview of the review process

Given below is an overview of the review process, which consists of 5 main steps. Each framework will be analysed taking into account the following criteria (Fig. 2).

A study carried out by Arns et al. [38] was able to uncover unique brain activation patterns in dyslexic children. A total of 38 participants: 19 dyslexics (11 males and 8 females) and 19 controls (11 males and 8 females) between the ages of 8–16 years took part in this study. The exclusion criteria included mental illness or genetic disorders in person or family history, neurological disorder, brain injury, addiction to drug or alcohol and serious medical conditions. The EEG data were acquired at a sampling rate of 500 Hz using the internationally recognised 10–20-electrode positioning system having 28 channels, namely Fp1, Fp2, F7, F3, Fz, F4, F8, FC3, FCz, FC4, T3, C3, Cz, C4, T4, CP3, CPz, CP4, T5, P3, Pz, P4, T6, O1, Oz and O2. The experiment was performed in a sound- and light-attenuated room, which was controlled at a room temperature of 22 °C. The EEG data were recorded for 2 min while being seated with eyes open, focusing the attention on a red dot displayed on a computer screen. The group of participants with dyslexia was also given few language tests. These tests consist of articulation, rapid naming of letters, phoneme deletion and spelling. These reading-related tasks were collected to find the correlation between EEG and the neurological findings of dyslexia. However, EEGs were not recorded while these tasks were performed; instead, the above-explained tasks with eyes open were used since the EEG of resting state highly correlated with the tests.

The data are EOG-corrected prior to the analysis. These data are then examined using the power spectral analysis. The approach followed is that the data are first partitioned into adjacent 4-s sections, next the data are transformed to the frequency domain from the time domain using fast Fourier transform (FFT), and finally the average power spectra are calculated for specified frequency bands ranging within the delta, theta, alpha and beta bands. The EEG data are then analysed statistically using one-way ANOVA to find the significant differences between the dyslexic and control group. Further, a correlation matrix is acquired for correlations between the variables within the dyslexic group. The significant measures of the EEG power and coherence data obtained from the two groups are submitted for the correlation analysis with the four language tests explained above. The study revealed that the dyslexic group had increased slow theta and delta activity in the frontal and right temporal areas of the brain. Beta was clearly increased at F7, and significant correlations were found between the EEG coherence and the dyslexia tests [38] (Table 1).

**Table 1:** Overview of the review process significant correlations for frequency bands versus dyslexia tests [38]

	Location versus subtest	Correlation and sign		
Delta	C4-C3 versus ART	*r* *=* 0.568	*df* *=* 17;	*P* *=* 0.017
	T4-FC4 versus ART	*r* *=* 0.508	*df* *=* 17;	*P* *=* 0.037
	C4-T4 versus ART	*r* *=* 0.527	*df* *=* 17;	*P* *=* 0.030
	T3-FC3 versus ART	*r* *=* 0.541	*df* *=* 17;	*P* *=* 0.025
	T3-FC3 versus PD	*r* *=* 0.520	*df* *=* 17;	*P* *=* 0.033
	C3-F7 versus RNL	*r* *=* 0.638	*df* *=* 17;	*P* *=* 0.006
	C3-Fp1 versus RNL	*r* *=* 0.662	*df* *=* 16,	*P* *=* 0.005
	CP4-F8 versus RNL	*r* *=* 0.527	*df* *=* 18;	*P* *=* 0.025
	FC3-F7 versus RNL	*r* *=* 0.576	*df* *=* 17;	*P* *=* 0.015
	T4-F8 versus SPL	*r* *=* 0.529	*df* *=* 17;	*P* *=* 0.029
	CP4-T4 versus SPL	*r* *=* 0.491	*df* *=* 17;	*P* *=* 0.045
Theta	C3-F7 versus RNL	*r* *=* 0.598	*df* *=* 17;	*P* *=* 0.011
	FC3-Fp1 versus RNL	*r* *=* 0.772	*df* *=* 16;	*P *< 0.000
	T3-FC3 versus ART	*r* *=* 0.527	*df* *=* 17;	*P* *=* 0.030
	C3-T3 versus ART	*r* *=* 0.532	*df* *=* 17;	*P* *=* 0.028
Alpha	T4-FC4 versus RNL	*r* *=* 0.576	*df* *=* 17;	*P* *=* 0.015
	T4-FC4 versus PD	*r* *=* 0.653	*df* *=* 17;	*P* *=* 0.005
	C4-T4 versus RNL	*r* *=* 0.508	*df* *=* 17;	*P* *=* 0.038
	C4-T4 versus PD	*r* *=* 0.565	*df* *=* 17;	*P* *=* 0.018
Beta	C4-T4 versus RNL	*r* *=* 0.501	*df* *=* 17;	*P* *=* 0.041
	C4-T4 versus SPL	*r* *=* 0.617	*df* *=* 17;	*P* *=* 0.008
	C4-T4 versus PD	*r* *=* 0.602	*df* *=* 17;	*P* *=* 0.011
	CP4-T4 versus RNL	*r* *=* 0.521	*df* *=* 17;	*P* *=* 0.032
	CP4-T4 versus SPL	*r* *=* 0.637	*df* *=* 17;	*P* *=* 0.006

*ART* articulation, *PD* phoneme deletion, *RNL* rapid naming letters, *SPL* spelling

This study only performs statistical analysis using the EEG data and does not present any classification mechanisms to differentiate between dyslexics and non-dyslexics. The data collection has been carried out wisely, taking into account an equal number of participants, a sufficient number of EEG channels, excluding criteria that could have an effect on the brainwave recordings and by collecting the data in a consistent and suitable environment. However, since the EEG data are collected only in the resting state and not while the tests are actually being undertaken, important artefacts specific to each task are most likely to be missed out. Since the EEGs were recorded only in the resting state, the only main unwanted artefact being the eye blinks has been removed in the pre-processing step of the analysis. The input features using the EEG recordings include the power spectra for specified frequency bands such as alpha, beta and theta at each EEG channel. One of the significant findings being the increase in beta frequency verifies that the brainwaves get activated significantly in dyslexics while performing tasks, in this case specifically reading-related tasks.

A framework for detecting abnormalities in dyslexia using approximate entropy of EEG signals was proposed by Andreadis et al. [39]. Approximate entropy (ApEn) is a ‘statistical parameter used to quantify the regularity of a time series data of physiological signals’ [39]. This study consisted of a total of 57 participants: 38 dyslexics (26 males and 12 females) and 19 controls (7 males and 12 females) between the ages of 2–13 years. The exclusion criterion comprises difficulties in hearing, history of head injury, neurological diseases or attention deficit disorders.

The EEG for this study was recorded using the international 10–20 system, containing 15 channels, namely Fp1, F3, C5, C3, Fp2, F4, C6, C4, O1, O2, P4, P3, Pz, Cz and Fz. The experiment for this study is that a single sound tone was presented to the participant via earphones, which was of a high frequency of 3000 Hz or low frequency of 500 Hz, followed by numbers that had to be memorised. The brainwave data were collected as EEG signal for 500 ms before the stimulus and as event-related potential (ERP) after the stimulus for 1000 ms.

The pre-processing mechanisms used in this study include two main steps. The first step was recording the electrooculography (EOG) and rejecting values higher than 75 μV, and the second step was normalising the waveforms by subtracting the mean value and dividing by the standard deviation of each signal. These data are then analysed using ApEn and Cross-ApEn (comparing EEG signals from two electrodes). A support vector machine (SVM) classifier was then implemented using the statistical significant electrodes for all subjects obtained using ApEn as input features. This classifier offered promising results achieving a sensitivity of 89.47% and specificity of 57.89%. The study was then taken a step forward to enhance the classifier using the input features from Cross-ApEn. This method looks at significant pairs of electrodes instead of evaluating electrodes on its own. Although this technique delivered better discrimination abilities, no clear pattern has yet been found because there were a very high number of statistically significant pairs of electrodes.

Looking at the study as a whole, it can be stated that the researchers have been able to successfully develop a classifier that can differentiate between the dyslexic and the non-dyslexic. However, the experiment used looks into only the working memory abilities and does not involve any reading- or writing-related elements. Since dyslexia is a condition that causes deficiencies in reading and writing abilities, important factors required for the differentiation process could be missed out. The same research team performed another analysis using the same experiment and data by using wavelet entropy [40]. The findings revealed that wavelet entropy could be used as a quantified measure to observe and analyse EEG and ERP signals to detect brain patterns specific to dyslexia.

A Malaysian research team conducted a frequency analysis of EEG signals generated between dyslexic and normal children during writing [41, 42]. The EEGs were recorded from a total of 6 right-handed children: 3 dyslexic and 3 control subjects between the ages of 8–12 years using the standard international 10–20 system. This study uses only 4 EEG channels, namely C3, C4, P3 and P4. The experiment involved collecting EEGs in the relaxed state and while performing writing-related activities, which were designed based on the conventional method of diagnosing dyslexia.

During the pre-processing phase, unwanted artefacts being electrocardiograms (ECG) and electrooculogram (EOG) were filtered out. Next, the signals containing the writing-related data were extracted using a band-pass FIR filter ranging from 8 to 30 Hz. For the frequency analysis, the signals are transformed to the frequency domain from the time domain using fast Fourier transform (FFT). The study revealed that the dyslexic children consume more energy which results in high-frequency beta wave relaxed states during writing-related activities compared to normal children. The frequency range identified for dyslexic children is between 22 and 28 Hz, whereas for non-dyslexic children it is between 14 and 22 Hz (Tables 2, 3).

**Table 2:** Frequency range (Hz) of EEG for relaxed state [42]

Electrode	Dyslexic children	Normal children
C3	9–12	9–10
C4	10–12	9–10
P3	9–12	9–10
P4	10–12	9–10

**Table 3:** Frequency range (Hz) of EEG for writing activities [42]

Electrode	Dyslexic children		Normal children	
	Alpha sub-band	Beta sub-band	Alpha sub-band	Beta sub-band
C3	9–10	23–27	9–10	15–22
C4	9–10	22–27	9–10	15–20
P3	9–10	23–26	9–10	14–18
P4	9–10	22–28	9–10	14–20

Overall, this study does not provide any classification mechanism. It only analyses the frequencies obtained from the dyslexic and non-dyslexic groups. Looking at the number of channels and the number of participants used for the study, it can be implied that the numbers are too small to arrive at a conclusion for using these results for a framework to discriminate between the dyslexic and the non-dyslexic. The study has explicitly used subjects that are right-handed, which is in fact an important factor since the handedness has an effect on the EEG activities between the right-handed and left-handed subjects [43, 44]. However, excluding factors that could have an effect on the EEG recordings has not been taken into consideration. Additionally, it is not indicated whether a silent and temperature-controlled room was used to carry out the experiment. The pre-processing techniques used in this study are similar to previous similar studies; however, since this study involves hand movements, it is not specified how the artefacts generated from the hand movements were filtered out. Further, the experiment focuses only on the writing-related tasks.

Frid, Breznitz [45] proposed a support vector machine (SVM)-based algorithm for differentiating between dyslexic readers and regular readers using ERPs. The study was carried out with a total of 50 participants: 20 dyslexics and 30 controls of the ages between 24 and 40 years. The signals were recorded at a sampling rate of 2048 Hz using the standard 10–20 system with 64 channels. The experiment used in the study is that the subject is required to press a button in response to a target stimulus, which is a tone. The conditions consist of 50 stimuli of target tones at frequencies of 1000 Hz and 50 non-target tones of 2000 Hz.

The data collected is first pre-processed using a band-pass filter at 0.1–100 Hz, and then a notch filter at 50 Hz is used to remove noise caused by electric power lines, and finally unwanted artefacts such as eye and muscle movements are filtered out. The next step is the feature selection where the features with the most relevance and the ability to discriminate are chosen. The five features selected are positive area (Ap), maximal peak amplitude/time ratio (Mp), spectral flatness measure (SFM), standard deviation and skewness, and power spectral density (PSD). Although the classification was first attempted using a single classifier for all features, it was not successful. Therefore, the approach follows was to use ensemble SVMs. The classification results were compared for the combinations: the best single feature, an ensemble of three SVMs and only the left or right hemispheres.

To recapitulate, the study uses a simple experiment task, which relates to working memory and reasoning abilities, but does not engage any stimulus with regard to reading or writing which are important factors in detecting unique patterns to dyslexia. This may have bypassed on activating vital areas of the brain specific to dyslexia. The study does not indicate whether they were any inclusion and exclusion criteria taken into account when recruiting the participants, which could increase the likelihood of having outliers within the groups selected.

A classification model to distinguish dyslexic children from the normal children during rest state was suggested by [46]. A total of 6 participants: 3 dyslexics and 3 controls within the ages of 4–7 years took part in this study. The EEG data are collected using the international 10–20-electrode placement system using 8 channels with a sampling rate of 250 Hz. The experiment is carried out in a room with controlled temperature and lighting while the participants are in the resting state with both eyes closed and eyes open.

During the pre-processing phase, noise and irrelevant artefacts have been removed. Since the data collection is done in the resting state, the frequency band relating to this state is alpha, and this has been extracted using band-pass filtering. The next phase being the feature extraction is performed using kernel density estimation (KDE), which is an artificial neural network technique organised in several different layers [46]. Finally, the classifier is trained using multilayer perceptron (MLP). This mechanism was able to obtain an accuracy rate of 90% to classify the dyslexic and non-dyslexic during both eyes open and eyes closed conditions.

To wrap up, the study uses EEG data from only the resting state disregarding the essential reading- and writing-related brainwave data. The number of participants and the number of channels used are quite low compared to previous similar research [39, 45]. No inclusion or exclusion criteria for participants used are indicated. Further, although the study gave a 90% accuracy rate since the data set used is very small it is very encouraging.

A wavelet packet analysis of EEG signals between dyslexic and non-dyslexic children during writing was proposed by [47]. A total of 8 subjects: 4 dyslexics and 4 controls between the ages of 7–12 years took part in this study. The EEG data were recorded in the temperature-controlled room at 24 °C using the international 10–20 system with 4 channels, namely C3, C4, P3 and P4, having a sample rate of 256 Hz. The signals were captured in the relaxed state, writing state and during letter recognition, and each task was repeated 6 times. This is then examined using wavelet packet analysis for alpha and beta frequency bands. The outcome of the study discovered that there was no significant difference in the alpha band frequencies during the relaxed state and writing state in dyslexics; however, for non-dyslexics the alpha band frequency was higher during relaxed state compared to writing state. During writing, beta frequency was higher in dyslexics compared to non-dyslexics.

This study looks into the brain behaviours during the resting and writing states, but does not look into the reading state. No information is provided about pre-processing the signal to remove unwanted artefacts such as eye blinks. The number of subjects and the number of channels used in the study are low compared to previous similar research [39, 45]. Finally, the study performs only as analysis and does not perform any classifications.

## Is there a need for an improved framework?

This section will examine all the frameworks as a whole and ultimately propose an improved framework.

### Data collection

#### Number of participants

There are many important decisions to be made prior to the data collection to make the experiment successful. One of the most important decisions to be made is to determine the number of participants required for the study. The review disclosed that some studies had too little subjects, which makes the outcome less reliable (Table 4).

**Table 4:** Determination of number of subjects

Research	Test group size	Control group size	Total
Different brain activation patterns in dyslexic children: Evidence from EEG power and coherence patterns for the double-deficit theory of dyslexia [38]	19	19	38
Wavelet entropy differentiations of event-related potentials in dyslexia [40]	38	19	57
Detecting complexity abnormalities in dyslexia measuring approximate entropy of electroencephalographic signals [39]	38	19	57
Comparison between characteristics of EEG signal generated from dyslexic and normal children [42]	3	3	6
An SVM-based algorithm for analysis and discrimination of dyslexic readers from regular readers using ERPs [45]	20	30	50
Classification of dyslexic and normal children during resting condition using KDE and MLP [46]	3	3	6
Wavelet packet analysis of EEG signals from children during writing [47]	4	4	8
Mean sample size (rounded)	18	15	32

In medical research, the number of subjects used for the study is mostly limited because of uniqueness, ethical considerations, time and cost. Therefore, it is important to identify the optimal sample size to avoid the sample being too small resulting in not being able to recognise important effects and the sample being too large resulting in a waste of resources. Using the sample size of a similar study is one of the approaches that can be used to determine the sample size [48]. In this case, instead of relying on one previous similar study, the sample size can be determined by getting the mean sample size of multiple similar studies. According to the calculation using past similar research, we can suggest having approximately 15 subjects for each group.

**Fig. 3:**
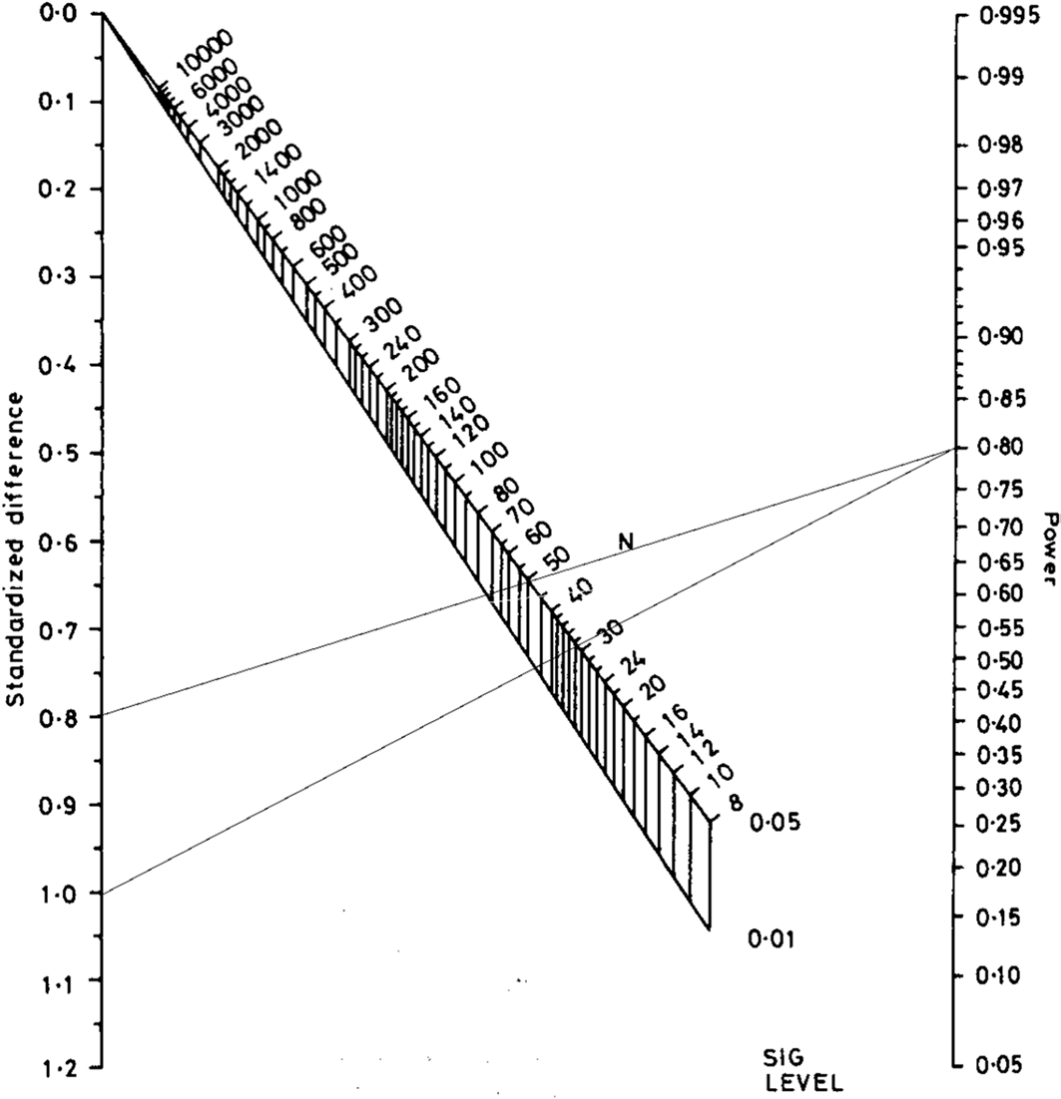
Altman’s nomogram sample size calculation [71]

Another technique to determine the number of subjects is the Altman’s nomogram sample size calculation. According to this calculation for a power of 0.80 (*P* value significance of 0.05) and a standardised difference value between 0.8 and 1.0 (Cohen’s d effect size), the total number of subjects would vary between 50 and 30 participants. Therefore, the number of subjects would per group vary between 25 and 15 (Fig. 3).

#### Age range

According to previous similar studies, EEG-based pattern classification frameworks for dyslexia studies have been carried out on children as well as adults, which means that the study can be used on either group. However, it is important to make sure that the subjects of age range selected have parallel reading and writing abilities (Table 5).

**Table 5:** Determination of age range

Research	Age range (years)
Different brain activation patterns in dyslexic children: evidence from EEG power and coherence patterns for the double-deficit theory of dyslexia [38]	8–16
Wavelet entropy differentiations of event-related potentials in dyslexia [40]	2–13
Detecting complexity abnormalities in dyslexia measuring approximate entropy of electroencephalographic signals [39]	2–13
Comparison between characteristics of EEG signal generated from dyslexic and normal children [42]	8–12
An SVM-based algorithm for analysis and discrimination of dyslexic readers from regular readers using ERPs [45]	24–40
Classification of dyslexic and normal children during resting condition using KDE and MLP [46]	4–7
Wavelet packet analysis of EEG signals from children during writing [47]	7–12

#### Gender

The past similar studies reviewed have not compared any brainwave patterns specific to gender. Therefore, for future work, the comparison between the female and male dyslexic brainwave patters is a gap to be filled.

#### Environment

The data collection location and its environment is a very important factor to be looked at when recording EEGs. Below given is a summary of typical environment extracted from the review and more suggestions. These factors are important to make sure no interference caused to the signals, the subjects are comfortable and are not distracted.

- Sound- and light-attenuated room.
- Temperature-controlled room—if subjects are perspiring, it could cause problems to the recordings.
- Any extra equipment in the room should be electrically quiet—this can be checked via a probe test for electromagnetic signals [49].

#### EEG recording system and channels

**Fig. 4:**
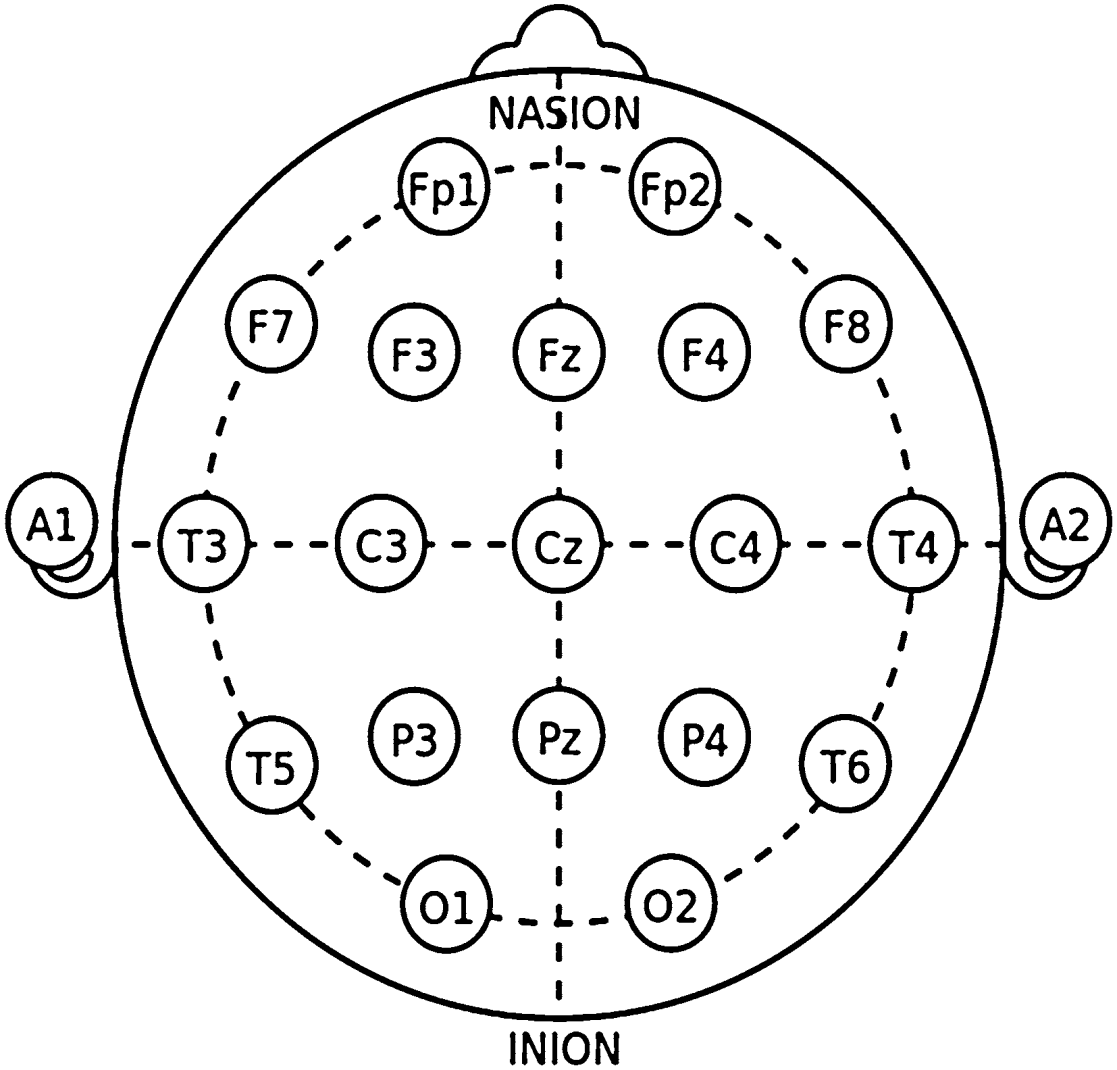
Arrangement of the international 10–20-electrode system [50]

The recommended electrode placement system is the international 10–20 system. This method describes the location electrodes on the scalp. The ‘“10” and “20” refer to the fact that the actual distances between adjacent electrodes are either 10 or 20% of the total front–back or right–left distance of the skull’ [50] (Fig. 4; Table 6).

**Table 6:** Popular choice of EEG channels

Research	Number of channels	Channels
Different brain activation patterns in dyslexic children: evidence from EEG power and coherence patterns for the double-deficit theory of dyslexia [38]	28	Fp1, Fp2, F7, F3, Fz, F4, F8, FC3, Fizz, FC4, T3, C3, Cz, C4, T4, CP3, Caps, CP4, T5, P3, PHz, P4, T6, O1, Oz, O2
Wavelet entropy differentiations of event-related potentials in dyslexia [40]	15	Fp1, F3, C5, C3, Fp2, F4, C6, C4, O1, O2, P4, P3, PHz, Cz, Fz.
Detecting complexity abnormalities in dyslexia measuring approximate entropy of electroencephalographic signals [39]	15	Fp1, F3, C5, C3, Fp2, F4, C6, C4, O1, O2, P4, P3, PHz, Cz, Fz.
Comparison between characteristics of EEG signal generated from dyslexic and normal children [42]	4	C3, C4, P3, P4
An SVM-based algorithm for analysis and discrimination of dyslexic readers from regular readers using ERPs [45]	64	F3, F4, P6, PHz, F8, CP4, AF7, F3, F5, T7, PO3, FC6, TP7, P7 (not all are given)
Classification of dyslexic and normal children during resting condition using KDE and MLP [46]	8	F3, F4, C2, C3, C4, P3, P4, T3, T4
Wavelet packet analysis of EEG signals from children during writing [47]	4	C3, C4, P3, P4
Popular EEG channels for identifying unique brainwave patterns for dyslexia	Fp1, F3, Fz, F4, F7, F8, T3, C3, Cz, C4, T4, PHz, AF3, TP7, P7	

The popular choice of EEG channel list was determined using channels specifically mentioned as prominent for classification in a study and channels that overlap at least between 2 studies.

#### Inclusion and exclusion criteria of the subjects

The inclusion and exclusion criteria summarised from the reviews are given below.

Exclusions:

- Mental illness.
- Genetic disorders in person or family history.
- Neurological disorders.
- Brain injuries.
- Drug or alcohol addiction.
- Serious medical condition.
- Difficulties in hearing/vision—this would not apply if the subject has corrected vision/hearing.
- Attention deficit disorders.

Inclusions:

- Handedness–the participants recruited need to be either left-handed or right-handed and not have a mix of the both. This is because there is a difference in EEG activities between the right-handed and left-handed subjects [43, 44].

### Experiment

As explained before, it is now understood that dyslexia is a disability that causes difficulties in reading and writing despite normal (or above) intelligence and sensory capabilities. Therefore, it can be presumed that dyslexia-specific brainwave activation patterns are more prominent during performing reading and writing activities instead of having tasks that are only related to the working memory and reasoning. Reading-related tasks can be drilled down further to find out brain signal patterns while reading regular words against nonsense words. Phonological awareness, ‘the ability to hear and manipulate the sounds’ in words [51], is one of the commonly found difficulties in dyslexics. Research [52] shows that dyslexics perform worse in reading irregular and nonsense words compared to regular words. Therefore, including a task to read nonsense words may show noticeable results. Today, writing is often replaced by typing in day-to-day activities; therefore, this too could be included in the tasks. Further, a task with a combination of reading and writing can be incorporated.

### Pre-processing

Pre-processing is one of the most important steps in the analysis process of the signals. This step makes sure unwanted artefacts are removed from the signal. When recording EEG signals, some of the most commonly seen irrelevant artefacts are the eye movements and eye blinks, and the common practices used for removing these from EEG signals are independent component analysis (ICA) and principal component analysis (PCA) [53, 54]. Comparison studies between these two techniques show that ICA produces better results compared to PCA [54, 55].

In addition, electrooculogram (EOG), which are produced from eye movements, and EEG recordings can contain contamination signals such as electromyogram (EMG) and electrocardiogram (ECG). Typically, body movements are kept to a minimum during EEG-based experiments. This is because movements cause unwanted artefacts in the EEG signal, making the analyses and classifications difficult. In fact, sometimes trials with unwanted artefacts are manually rejected from studies [56]. However, new methods have now been introduced making it possible to collect data during real-life activities instead of only collecting data during resting state or simple activities such as button clicks. Artefact subspace reconstruction (ASR) is one such method which can be used to filter out body movement and muscle burst artefacts from the EEG signals [57, 58]. ASR ‘relies on a sliding-window principal component analysis, which statistically interpolates any high-variance signal components exceeding a threshold relative to the covariance of the calibration data set. Each affected time point of EEG is then linearly reconstructed from the retained signal subspace based on the correlation structure observed in the calibration data’ [58].

**Fig. 5:**
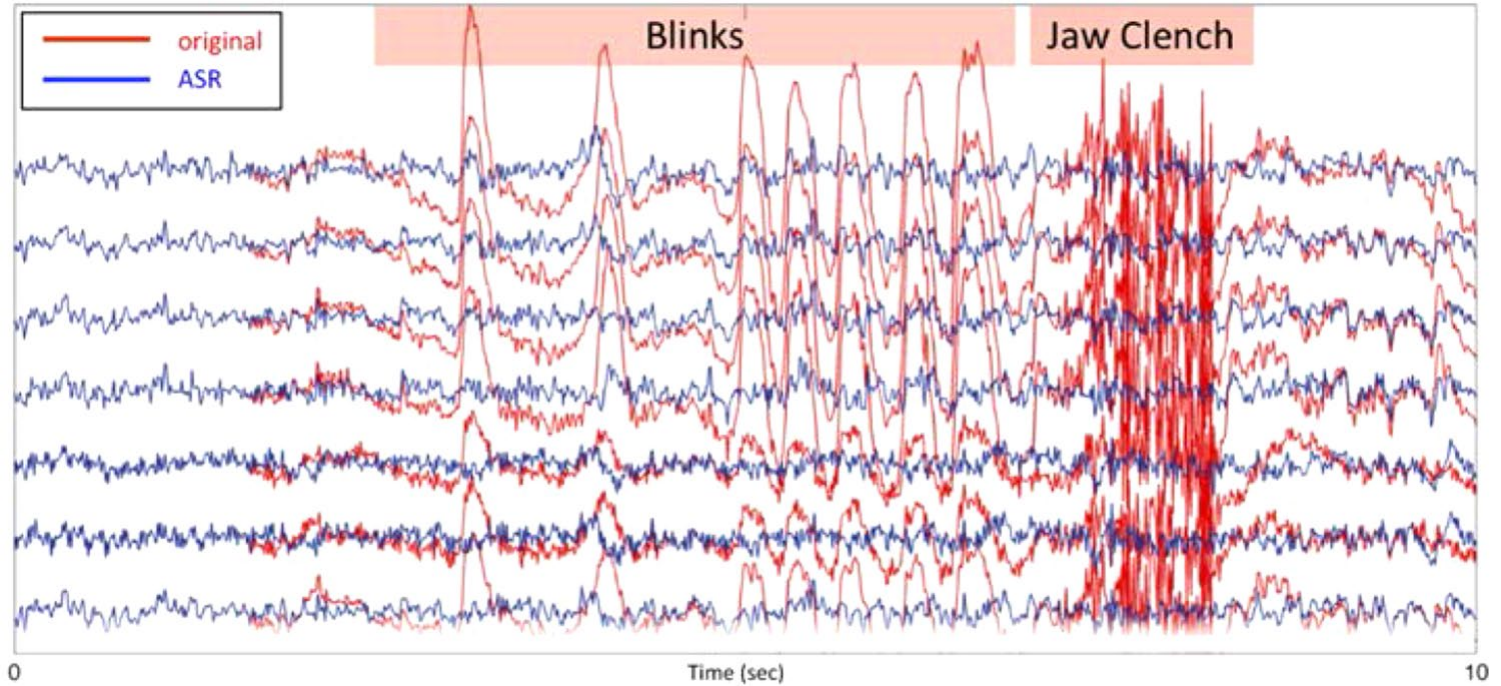
Example of filtering out movements from EEG using ASR [58]

ASR requires a 1-min EEG recording in the relaxed state, which is known as the calibration data set. This technique performs PCA on a sliding window, removes high variance up to three standard deviations above the mean and finally reconstructs using the remaining signal. This automated artefact removal technique is quite easy to use as it is available as a plug-in in EEGLAB (Fig. 5).

- state = asr_calibrate (calibrationData, samplingFrequency);
- cleanData = asr_process(experimentData, samplingFrequency, state);

Another important aspect to be filtered prior to the analysis is the noise caused by electric power lines. This is often seen at 60 or 50 Hz, and this can be filtered out using a notch filter.

### Analysis

#### Analysis method

There are mainly 2 types of analysis that could be used, which are namely frequency/Fourier analysis and wavelet analysis (Table 7).

**Table 7:** Analysis summary

Research	Analysis method
Different brain activation patterns in dyslexic children: evidence from EEG power and coherence patterns for the double-deficit theory of dyslexia [38]	Fast Fourier transform
Wavelet entropy differentiations of event-related potentials in dyslexia [40]	Wavelet entropy
Detecting complexity abnormalities in dyslexia measuring approximate entropy of electroencephalographic signals [39]	Approximate entropy and cross-approximate entropy
Comparison between characteristics of EEG signal generated from dyslexic and normal children [42]	Fast Fourier transform
An SVM-based algorithm for analysis and discrimination of dyslexic readers from regular readers using ERPs [45]	Time domain and frequency domain
Classification of dyslexic and normal children during resting condition using KDE and MLP [46]	Short-time Fourier transform
Wavelet packet analysis of EEG signals from children during writing [47]	Wavelet analysis

*Frequency analysis* One of the common analyses used in EEG-based pattern classification frameworks for dyslexia is the frequency analysis. The raw EEG signal recorded is in the time domain. This waveform is a combination of a number of sinusoidal waves although is it not directly visible. Fast Fourier transform, commonly known as FFT, can be used for the decomposition of the waveform into a sum of sinusoids of different frequencies. Therefore, by performing the FFT it helps detect spikes in the frequency domain which could not have been visible before.

*Wavelet analysis* This method decomposes a signal onto a set of basis functions called wavelets [59] and allows analysis on the frequency domain and time domain.

The analysis should be selected based on the expected outcome. Although wavelet gives extra information, this might not be important if the intension is only to identify which voltages are present at each frequency and not at what time the particular voltage was present. The decision for the analysis method is purely based on the experiment and expected outcome.

#### EEG sub-band decomposition

Once all the channels have been transformed to the frequency domain, this could be decomposed into sub-bands. Table 8 contains a summary of each frequency sub-band.

**Table 8:** EEG sub-band frequencies [68]

Frequency band name	Frequency bandwidth (Hz)	Usual human state associated with bandwidth	Example bandwidth
Delta	1–3.9	Deep sleep	
Theta	4–7.9	Drowsy, meditate	
Alpha	8–13.9	Relaxed	
Beta	14–29.9	Alertness, focused	
Gamma	30–64	Peak performance	

This method allows analysing the frequencies at specific frequency bands instead of analysing each frequency in isolation.

#### Feature extraction

The most important step in the analysis phase is the extraction of features. Feature extraction is transforming the input data into a set of features [34]. This helps to analyse the data in terms of a reduced set of features instead of the large original input data set. The input features identified through the review are power spectral density, entropy, positive area, maximal peak amplitude/time ratio, spectral flatness measure, standard deviation and skewness. Energy, average valley amplitude, peak variation, root mean square and power are few of the features used in recent EEG-related studies [31, 60, 61] that could be incorporated in EEG-based pattern classification for dyslexia frameworks as well. Adding all these features will not necessarily improve the validation accuracy; these features from other EEG studies are suggested so that these combinations could be tested and help improve dyslexia-based frameworks as it has helped improve other frameworks.

### Classification

The classification phase can be identified as the most important step in the dyslexia pattern identification process. Once all the data are ready, it is important to select the best classification algorithm. The popular choices of classification algorithms used in past similar research are support vector machine and multilayer perceptron. EEG classifications have also been performed for other conditions using classifiers such as fuzzy support vector [62], optimum-path forest classifier [31], linear discriminant analysis and neural networks [63]. Out of the choices below are 3 popular choices, along with pros and cons of each choice.

#### Linear discriminant analysis

Linear discriminant analysis classifies data by first creating ‘models of the probability density functions for data generated from each class. Then, a new data point is classified by determining the probability density function whose value is larger than the others’ [62]. The algorithm ‘assumes that each of the class probability density functions can be modelled as a normal density and that the normal density functions for all classes have the same covariance’ [62].

Linear component analysis is known to be a simple classifier that requires very small computations. However, this algorithm is not suitable for complex nonlinear EEG classifications since it does not produce good results for such scenarios [64].

#### Neural networks

Neural networks are ‘an assembly of several artificial neurons which enables to produce nonlinear decision boundaries’ [64].

Neural networks perform better for EEG classifications compared to linear discriminant analysis since it can be used to implement boundaries for nonlinear classifications. Nevertheless, to acquire the desired level of accuracy, it is important to choose a suitable number of hidden units, which can become problematic. Having a larger number of hidden units than required results in memorising the training set which causes poor generalisation [63].

#### Support vector machines

Support vector machine is a supervised learning method [65], which can handle both linear and nonlinear classifications. It produces a hyper-plane having the maximal margin to the support vectors. Support vector machine can classify even overlapping and non-separable data sets by mapping onto higher-dimensional spaces using the kernel functions [34, 63].

#### Popular classification technique

Through the comparison of the popular choices of the classification algorithms for EEG signals, it can be concluded that support vector machine (SVM) is a better choice.

SVM has been used in past research for many EEG signal classifications. Successful results have been obtained in classifying mental tasks [66], seizure detection [34, 35], discrimination between dyslexics and non-dyslexics [39, 45], epilepsy diagnosis [31], vigilance analysis [67], etc.

Further research [63, 64] has recommended support vector machines as a more appropriate choice for EEG signal classifications. Recent EEG-related studies [68–70] have been able to obtain good validation accuracies using SVM classifiers.

## Conclusion

Dyslexia is a disability with a neurological origin, affecting a significant amount of the population, which causes difficulties in reading and writing despite average intelligence. It is a heritable condition, but not a disease or defect that can be cured, rather a state that can he helped with proper targeted assistance. Research has shown distinctions in the brainwave patterns and brain structures of dyslexics compared to non-dyslexics (normal). Though dyslexia has a neurological origin, the conventional dyslexia detection techniques used are often based on behavioural aspects such as reading, writing, intelligence quotient (IQ) and memory abilities.

Many researches have attempted to introduce and improve EEG-based pattern classification frameworks for dyslexia. This review paper has identified pros and cons of existing frameworks. The frameworks are reviewed based on the criteria: data collection, pre-processing, analysis and classification. According to the review, it was revealed that frameworks require a minimum of 15 subjects per each group, the studies could be conducted on children or adults, and comparison between the female and male dyslexic brainwave patterns need to be conducted. It is also important to identify the inclusion and exclusion criteria prior to the data collection to minimise the number of outliers.

It was discovered that the experiments used were often simple tasks, which measure working memory and reasoning abilities instead of reading and writing abilities. This could be because to reduce the unwanted artefacts caused by body movements in the EEG signals during reading and writing activities. We have proposed using ASR a successful method that has been used in recent studies to filter out body movement and muscle burst artefacts from the EEG signals [57, 58]. Finally, we have proposed more input features and recommended SVM as the classifier to be used in EEG-based pattern classification frameworks for dyslexia.
